# Examining access to and trust in sources of COVID-19 information among CALD Asian communities in New Zealand

**DOI:** 10.1371/journal.pone.0319326

**Published:** 2025-03-28

**Authors:** Lynne Soon-Chean Park, Rebekah Jaung, Joohyun Justine Park, Changzoo Song

**Affiliations:** 1 School of Cultures, Languages and Linguistics, Faculty of Arts, University of Auckland, Auckland, New Zealand; 2 Te Kupenga Hauora Māori, Faculty of Medical and Health Sciences, University of Auckland, Auckland, New Zealand; 3 Interdisciplinary Centre for East Asian Studies, Goethe University Frankfurt, Frankfurt am Main, Germany; University of Otago, NEW ZEALAND

## Abstract

During the COVID-19 pandemic, effective crisis communication has been crucial yet challenging, especially for culturally and linguistically diverse (CALD) communities. This study explored how CALD Asian communities in New Zealand accessed and trusted various sources of COVID-19 information. A cross-sectional online survey with 1,267 Asian respondents was conducted in 2021. Findings revealed that participants engaged with diverse sources for COVID-19 information, despite delays in government efforts to provide linguistically appropriate information. Those without English as a first language tended to have access to fewer information sources. Ethnic group preferences varied: Indian and South East Asian groups favoured official channels (mainstream media and government websites), the Chinese group preferred messaging applications and workplaces, and the Korean group showed a preference for ethnic community media. Trust was higher in formal information sources than online platforms and personal networks. Increased trust was noted in social media among non-English speakers and messaging applications among Koreans, while decreased trust was seen in messaging applications among Chinese and workplace information among Koreans. This research underscores the necessity of multifaceted, linguistically, and culturally appropriate crisis communication strategies. It advocates for proactive measures to establish networks for distributing vital information to CALD communities during future crisis communication.

## Introduction

Since the outbreak of the Covid-19 pandemic in late 2019 through early 2023, governments worldwide have grappled with the imperative of effective crisis communication as part of the overall pandemic response. Crisis communication is defined as the management and dissemination of information in the face of sudden, high-risk events, like disease outbreaks [1]. Challenges arise not only in keeping information up to date but also in the recipients’ interpretation of that information, which becomes even more complicated when considering linguistic, health, and digital literacy [2–5].

Culturally and linguistically diverse (CALD) communities are a key population subgroup for whom generic crisis communication strategies are less likely to be effective. Marked racial and ethnic disparities in rates of hospitalisations and COVID-19 deaths have been reported across the globe [6]. This observation is particularly pronounced among CALD groups across multiple populations [7–10].

Within the domain of communication specifically, little is known about how effective generic crisis communication strategies have been in reaching and engaging with CALD communities. This research paper delves into the complexities surrounding access to and trust in government-provided COVID-19 information sources among CALD communities in New Zealand, with a particular focus on Asian communities. By doing so, this study aims to shed light on the challenges and opportunities inherent in crisis communication to CALD groups.

### CALD communities globally and in New Zealand

The term CALD encompasses a range of different cultural and linguistic backgrounds in communities with ‘diverse languages, ethnic backgrounds, nationalities, traditions, societal structures, and religions’ [11, par 6]. Using the term CALD, as opposed to other categorisations such as minorities, non-English speakers, or immigrant communities, embraces a broader and less stigmatising perspective. It refrains from imposing a minority or other status implicitly, thereby fostering a more equitable dialogue. It acknowledges the multifaceted nature of diversity by combining both cultural and linguistic aspects, allowing for a more comprehensive understanding of the unique challenges faced by these communities [11]. The term attempts to capture the heterogeneity within the communities without reducing their complexities to single labels and it focuses on characteristics which are specifically relevant to the domain of communication. Moreover, CALD as an umbrella term is beneficial for policy-making and research, as it consolidates diverse communities under a single term, facilitating more streamlined analysis and resource allocation [12].

However, the term CALD also carries certain limitations. Critics argue that the umbrella term, intended to be inclusive, can inadvertently become a tool for generalisation [11]. It risks diluting the unique challenges faced by individual communities under the broad banner of cultural and linguistic diversity. Nevertheless, the adoption of the term CALD in the New Zealand context could offer a more inclusive and encompassing approach to public health policies and communication strategies. By doing so, it may help to address some of the limitations evident in previous interventions, thereby fostering better engagement and trust among New Zealand’s CALD population. Moreover, the term CALD is particularly relevant when assessing the efficacy of crisis communication. By recognising a community as CALD, authorities can tailor COVID-19 resources that are not just linguistically appropriate but also culturally sensitive.

In New Zealand, Asian communities constitute a significant proportion of the wider CALD population [13]. Asian communities in New Zealand are diverse, comprising various nationalities, languages, and religious backgrounds, among other cultural elements [14]. While not relevant for all people with an Asian ethnicity, the CALD umbrella is one lens for understanding the unique challenges some members of Asian communities face, especially when it comes to accessing vital public information. This diversity is further exemplified when considering language proficiency. For instance, in the Northern region of New Zealand (comprising of Auckland and Northland), where 64% of the nation’s Asian population reside, the proportion of people who were able to have a conversation in English ranged from 75-90% depending on specific ethnic group [13]. This variability in English language proficiency is crucial to acknowledge, as experiences may diverge significantly even within the same ethnic group based on level of familiarity with English and cultural nuances. Therefore, recognising Asian communities as part of the broader CALD population is essential in focusing on customised strategies that are both linguistically appropriate and culturally sensitive, particularly during crisis like the COVID-19 pandemic.

### Crisis communication for CALD communities during the COVID-19 pandemic

For CALD communities, the challenges related to emergency communication are even more pronounced. Language barriers and limited access to translated government information create significant hindrances [2,3]. For example, in the United States, linguistic barrier of CALD communities was associated with a higher incidence of COVID-19 complications [3]. In the United Kingdom, studies have demonstrated that CALD communities like the African-Caribbean, Somali, and South Asian groups have faced similar challenges in accessing comprehensible health information [8].

Moreover, delays in translations and a scarcity of multilingual resources are associated to miscommunication and the risk of medical errors [4]. In Australia, organisations have raised concerns about the absence of accessible and reliable COVID-19 information in languages other than English, specifically for CALD communities in public housing towers. They emphasised that the Australian Government’s rapid response to COVID-19, unfortunately, left these communities as an afterthought without adequate access to COVID-19 information, potentially making them more vulnerable than other parts of the Australian community [5]. This issue extends beyond mere translation concerns to encompass inclusivity in decision-making and crisis communication strategy.

Trust issues are also intricately tied to crisis communication for CALD communities. For example, CALD communities in the United States have shown long-standing mistrust in healthcare and governmental systems, making crisis communication more challenging [2]. Similarly, in Europe, community members preferred receiving information from trusted individuals within their community, suggesting that established public communication channels are not always effective in a certain CALD contexts [8].

The role of trust in health information sources is paramount for enabling individuals to effectively interpret and respond to disseminated information [15,16]. Trust becomes particularly indispensable in the context of pandemic response strategies aimed at CALD communities [17]. Within these communities, prevalent miscommunication during the COVID-19 pandemic has been largely attributed to a decline in trust and credibility towards government authorities [18]. Such a deficiency in institutional trust has been notably prevalent and has contributed to racial disparities in health outcomes [19].

Moreover, familiarity also facilitates the dissemination of health information. Individuals who are trusted within CALD communities, particularly those associated with settlement-support organisations and health services, wield considerable influence over the perceived credibility of disseminated health information [2]. Considering the high levels of trust and accessibility of General Practitioners (GPs) within CALD communities, these medical professionals become crucial channels for effective health information dissemination.

Consequently, it becomes imperative for governments and crisis management teams to prioritise strategies that identify and utilise trusted channels and individuals for effective crisis communication. Employing a multi-dimensional approach is crucial in this context. This approach is not only essential for breaking linguistic and health barriers but also for deeply rooting public messaging in the cultural and social fabrics of CALD communities. A multi-dimensional approach recognises the diverse needs and characteristics within these communities, considering factors including language, culture, age in the content and delivery of information and addressing them through various channels and methods to ensure inclusivity and effectiveness. In parallel, such a holistic approach in which effective communication is delivered in a coordinated manner alongside other aspects of crisis management is equally important. It significantly contributes to diminishing healthcare information disparities and fostering enhanced, timely communication [5]. By adopting a holistic approach, the strategy incorporates a broader perspective, considering not only the direct impact of communication but also its longer-term effects on community well-being and trust in public health systems. This comprehensive strategy ensures that the communication is not only disseminated effectively but is also receptive and responsive to the diverse needs of the community.

### Communication during the pandemic in New Zealand

New Zealand has been relatively successful in deploying a communication strategy that has won commendation for its efficacy in disseminating information and fostering social cohesion [20]. This communication strategy sat alongside an early investment in a national elimination approach to COVID-19, which was comparatively radical, including the near complete closure of borders, lockdowns, and a comprehensive case and contact tracing system [21,22].

New Zealand’s approaches during the first two years of the pandemic resulted in outcomes including low COVID-19 mortality rates and increased life expectancy [23]. A key medium of the New Zealand government’s communication strategy was regular televised briefings featuring sign language interpreters to ensure accessibility [20]. These briefings were held daily for most of the first two years of the pandemic and were accompanied by question time with the media. These sessions formed the basis of most reporting about the day-to-day progress of the pandemic and pandemic response at that time [24].

Additionally, the dissemination of information from the briefings and relevant policy documents utilised a multiple-platform approach, including traditional media, digital platforms, and social media channels. A number of strategic frameworks and metaphors were employed by the New Zealand government to effectively convey not only daily case numbers and policy changes but also messages of motivation and appeals for cooperation. Two key frameworks, such as ‘the team of five million’ and ‘go hard and go early,’ were frequently reiterated in the Prime Minister’s daily briefing speeches and often drew upon competition and sports imagery, resonating positively with aspect of national identity for many New Zealanders [20,25]. Alongside these, the government’s presentation of COVID-19 policies during the early stages of the pandemic was also characterised by the four-part ‘Alert Level System’ [21] and the metaphor of ‘the bubble’ [25]. These models allowed information to be communicated in both text and visual forms, and works such as ‘the bubble,’ created in collaboration by microbiologist Dr Siouxsie Wiles and cartoonist Toby Morris, have even been used internationally to make public health and scientific concepts more accessible to broad audiences [26].

### New Zealand government’s approach for CALD communities

In contrast to the prominent level of attention given to general communication as part of the pandemic response, there are few reports of how local CALD communities were specifically considered during these efforts. Focusing on the government’s COVID-19 website, ‘United Against COVID-19,’ translated materials were easy to locate. However, the quality and range of information were low compared to what was available in English, and were not adequate for keeping communities informed [27].

Key areas of weakness in the communication approach for CALD communities include: timeliness of information, inconsistencies on what resources were available in what languages, and a lack of clarity about when resources had been created, a vital piece of context in the ever-changing policy context of the early pandemic response [27]. The limitations in linguistically appropriate COVID-19 resources produced by the New Zealand government were only highlighted in a public forum in March 2021 after the Prime Minister had criticised a person who was part of COVID-19 cluster (which included members of a CALD Asian community) for not following the rules during one of the daily briefings [28,29]. Prior to this time, timely translated information was largely provided on an informal basis by community groups and volunteers [30] rather than being coordinated and resourced by the government. This made it difficult for individuals to assess the reliability and usefulness of culturally and linguistically appropriate information. Additionally, the availability of such information often depends on the size and privilege of different CALD communities.

Against this backdrop, this study aims to examine the diverse information sources utilised by CALD Asian communities in New Zealand for accessing COVID-19 information provided by the government. Moreover, the study will assess the levels of trust that these communities place in each of these various information sources. By scrutinising these communication channels and evaluating the levels of trust accorded to them, the study will contribute to a more holistic understanding of the complex landscape of health communication in crisis contexts, focusing on the unique challenges and opportunities that arise within New Zealand’s CALD Asian communities. In doing so, this study will offer insights into potential improvements in public health messaging strategies tailored specifically for New Zealand’s CALD communities.

## Method

### Positionality

In New Zealand, all research must be considered through the lens of te Tiriti of Waitangi (the Treaty of Waitangi) - the foundational agreement between the Indigenous Māori and settlers – as part of best research practice [31]. As a research team, we recognise the multiple positions we hold as members of different Asian communities in New Zealand alongside our other identities. We understand that Asian peoples can also become tangata Tiriti (treaty people) in relation with Māori, and strive to uphold our Tiriti responsibilities as researchers on this land.

Regarding the value and limitations of the term ‘Asian’ we note that although it is sometimes pragmatic or strategic to come together as ‘Asian,’ this group is made up of many communities and individuals who have different understandings and relationships with ‘Asianness’ as an ethnic identity. As we describe the findings of our survey, we are mindful of the impossibility of trying to circumscribe a singular Asian experience of the pandemic.

Additionally, we acknowledge the risk of framing discussions about CALD Asian population in a deficit-focused discourse that assigns additional ‘need’ to CALD communities. However, we posit that CALD communities have a right to access vital public health information, as supported by the New Zealand Code of Health and Disability Services Consumers’ Rights [32, see Right 5]. CALD communities along with all users of the New Zealand health and disability system have a ‘right to effective communication’ which puts the responsibility on service providers to ensure effective communication.

### Procedure and participants

Eligible participants were defined as people who were 16 years or older and self-identified as Asians living in New Zealand at the time of the survey. People who self-identified as members of the Asian ethnic group, according to Statistics New Zealand, made up the study’s target population. At the time of the 2018 New Zealand Census, 707,598 people self-identified as belonging to the Asian ethnic group, accounting for 15.1% of the country’s population [33].

To ensure ethical conduct, approval was granted by University of Auckland before recruitment began. Recruitment of participants was conducted using non-probabilistic purposive and snowball sampling techniques [34,35], just prior to the commencement of the Auckland lockdown, between August and September of 2021, a period during which the delta variant of the Coronavirus was dominant worldwide. These non-probabilistic methods were chosen due to time and budget limitations. The urgency to gather data during the COVID-19 period necessitated a rapid recruitment process, which was feasible through purposive and snowball sampling. To reach a broader section of the targeted population across New Zealand, the research team utilised various advertising methods, including social media platforms like Facebook and Instagram, community networks, online channels, and Asian community-related groups.

Before the survey was released to the public, a pilot study was conducted with 10 participants to provide feedback on the survey. Based on this feedback, the research team made the necessary adjustments to the survey. Given the diversity within the Asian population and the fact that many did not have English as their first language, the survey was translated into three frequently spoken Asian languages (Simplified Chinese, Korean, and Japanese). Professional translators translated the survey, and then nine bilingual reviewers reviewed the functional equivalence of the translation. Any necessary adaptations were made based on this feedback. The participant information sheet, which clarified the aims and design of the research, was provided at the beginning of the survey for informed consent, and participants were given the option to select their preferred survey language.

### Measures

The assessment of sources for COVID-19 information provided by the New Zealand government was conducted by adapting a question from the COVID-19 Health and Well-being Survey [36]. Participants were asked to select their personal information source(s) they used to access information on ‘the Government 1 pm Covid-19 briefing’ among the following nine options: *mainstream New Zealand media, local ethnic community media, New Zealand government websites, social media, search engines, messaging applications, family, whānau or friends, workplace,* and *other*. They were also given the choice to select a response option *not applicable*. Additionally, this study measured trust levels attributed to each of the nine sources of government health information. Participants were asked to reflect their previous experiences accessing information about COVID-19 during lockdown periods and rate their levels of trust for each source on a five-point scale (1 =  *I have no trust at all*, 2 =  *I have little trust*, 3 =  *I have some trust*, 4 =  *I have lots of trust*, 5 =  *I have complete trust*). Socio-demographic information was gathered from questionnaires about gender, age, ethnicity, first language, residential area, visa status, student status, and experience of working as an essential worker during the lockdown periods.

### Data analysis

Data analysis was performed using Stata/IC (version 16, StataCorp, College Station, TX). The study included nine data sources, but “other sources,” used by only 30 participants (2.4% of respondents), had a much lower usage rate than the other eight. Consequently, it was excluded from descriptive summaries and further analyses. Descriptive summaries for information source and trust of source were analysed by: gender (*female, male, another gender*), ethnicity (*Chinese, Indian, Korean, Southeast Asian, Other Asian*), age group (*16-29, 30-59, ≥ 60*), English as first language (*yes/no*), visa status (*temporary/permanent*), student status (*secondary, tertiary, not a student*), essential worker status (*yes/no*), and region (*Auckland/other, urban area/other*). The association between English as a first language status and ethnic grouping and use of different data sources (*used/did not use*) was examined using logistic regression, and the association between these independent variables and trust in different information sources (five-point scale) was examined through linear regression. We adopted linear regression analysis because the five-point Likert scale used to measure trust in information sources is widely regarded as approximating equal-interval scales, which justifies the use of parametric methods like linear regression [37]. The analyses for English as a first language included age, student status, ethnicity and gender as potentially confounding variables. The analyses for ethnicity included age, student status, gender and English as a first language status as potentially confounding variables.

Ethnicity grouping was informed by the Ministry of Health ethnicity protocols [38,39]. When required for data analysis, prioritised ethnicity according to Ministry of Health protocols was also derived [39]. All prioritised ethnic categories used in data analysis are level 2 categories of the protocols, except for Koreans. Although Koreans are part of the ‘other Asian’ group, their high representation (33.5%) among participants could disproportionately influence this category. Categorical data were described using count and proportion, and group difference was analysed using chi-square tests.

## Results

Of the 2,204 initial responses, we removed responses in which only the socio-demographic questions were answered (742 responses). Next, 10 responses which did not meet the recruitment criteria were deleted. From the remaining 1,452 responses, 185 responses had incomplete data related to the variables of interest (access to and trust in COVID-19 information sources). The socio-demographic profile of all 1,452 eligible participants, including those with incomplete survey responses, is outlined in a previous publication [40]. The current analysis included a total of 1,267 responses.

### Use of information sources

Table 1 and 2 show the overall access to COVID-19 information among respondents and by sociodemographic subgroup. Mainstream New Zealand media was the most frequently used source, followed by social media, and New Zealand government websites. Less than half of respondents used other sources such as family and friends, search engines, workplace, local ethnic community media, and messaging applications.

**Table 1:** Sources of COVID-19 Information by Demographic Grouping.

		Mainstream NZ Media	Local ethnic community media	NZ Govt websites	Social Media	Search Engines	Messaging applications	Family and friends	Workplace
**Total**	**N = 1,267**	**1,005 (79%)**	**277 (22%)**	**651 (51%)**	**700 (55%)**	**342 (27%)**	**275 (22%)**	**410 (32%)**	**315 (25%)**
*Gender*		0.429	0.250	0.066	0.342	0.160	0.317	0.852	0.275
Male ^a^	n = 380	293 (77%)	92 (24%)	177 (47%)	208 (55%)	95 (25%)	88 (23%)	104 (27%)	88 (23%)
Female^b^	n = 869	698 (80%)	183 (22%)	463 (53%)	479 (55%)	239 (28%)	181 (21%)	301 (35%)	220 (25%)
Other^c^	n = 18	14 (78%)	2 (11%)	11 (61%)	13 (72%)	8 (44%)	6 (33%)	5 (28%)	7 (39%)
*Age*		0.000	0.000	0.000	0.000	0.000	0.056	0.000	0.000
16-29^d^	n = 420	368 (88%)	33 (8%)	250 (60%)	307 (73%)	175 (42%)	86 (20%)	185 (44%)	113 (27%)
30-59	n = 759	586 (77%)	202 (27%)	370 (49%)	372 (49%)	154 (20%)	161 (21%)	205 (27%)	197 (26%)
60 and over	n = 88	51 (58%)	42 (48%)	31 (35%)	21 (24%)	13 (15%)	28 (32%)	20 (23%)	5 (6%)
*Prioritised ethnicity*		0.000	0.000	0.000	0.000	0.000	0.000	0.033	0.000
Chinese^e^	n = 362	293 (81%)	78 (22%)	194 (54%)	211 (58%)	113 (31%)	108 (30%)	131 (37%)	108 (30%)
Indian^f^	n = 133	105 (79%)	5 (4%)	83 (62%)	80 (60%)	44 (33%)	17 (13%)	30 (23%)	37 (28%)
Korean^g^	n = 424	307 (72%)	175 (42%)	145 (34%)	165 (39%)	59 (14%)	102 (24%)	134 (32%)	67 (16%)
SouthEast Asian^h^	n = 224	202 (91%)	10 (4%)	156 (70%)	164 (73%)	89 (40%)	28 (13%)	80 (36%)	69 (31%)
Other Asian^i^	n = 124	98 (79%)	9 (7%)	73 (59%)	80 (65%)	37 (30%)	20 (16%)	35 (28%)	34 (28%)

*Note*. The shaded boxes indicate where the difference in proportions within a subgroup category were statistically significant. Reference groups: ^a^ not male, ^b^not female, ^c^not another gender, ^d^other age categories (ordinal variable), ^e^ Non-Chinese, ^f^ Non-Indian, ^g^ Non- Korean, ^h^ Non-Southeast Asian, ^i^Not-Other Asian (nominal variable).

**Table 2:** Sources of COVID-19 Information by Social Grouping.

		Mainstream NZ Media	Local ethnic community media	NZ Govt websites	Social Media	Search Engines	Messaging applications	Family and friends	Workplace
First language		0.000	0.000	0.000	0.000	0.000	0.054	0.000	0.000
Other^a^	n = 904	675 (75%)	259 (29%)	399 (44%)	442 (49%)	179 (20%)	209 (23%)	263 (29%)	195 (22%)
English^b^	n = 363	330 (91%)	18 (5%)	252 (69%)	258 (71%)	163 (45%)	66 (18%)	147 (41%)	120 (33%)
*Visa status*		0.089	0.065	0.006	0.201	0.017	0.374	0.733	0.004
Temporary^c^	n = 248	187 (75%)	65 (26%)	108 (44%)	146 (59%)	52 (21%)	59 (24%)	78 (31%)	44 (18%)
Permanent^d^	n = 1019	818 (80%)	212 (21%)	543 (53%)	554 (54%)	290 (28%)	216 (21%)	332 (33%)	271 (27%)
*Student status*		0.025	0.000	0.000	0.000	0.000	0.266	0.000	0.012
High-school^e^	n = 39	35 (90%)	3 (8%)	20 (51%)	26 (67%)	19 (49%)	7 (18%)	22 (56%)	3 (8%)
Tertiary^f^	n = 296	247 (83%)	38 (13%)	186 (63%)	213 (72%)	119 (40%)	74 (25%)	120 (41%)	65 (22%)
Not a student^g^	n = 932	723 (78%)	236 (25%)	445 (48%)	461 (49%)	204 (22%)	194 (21%)	268 (29%)	247 (27%)
*Essential worker*		0.243	0.365	0.706	0.942	0.263	0.704	0.227	0.000
Yes^h^	n = 450	365 (81%)	92 (20%)	228 (51%)	248 (55%)	113 (25%)	95 (21%)	136 (30%)	181 (40%)
No^i^	n = 817	640 (78%)	185 (23%)	423 (52%)	452 (55%)	229 (28%)	180 (22%)	274 (34%)	134 (16%)
*Regional group 1*		0.772	0.000	0.035	0.003	0.131	0.619	0.769	0.046
Other^j^	n = 328	262 (80%)	45 (14%)	185 (56%)	204 (62%)	99 (30%)	68 (20%)	104 (32%)	95 (29%)
Auckland^k^	n = 939	743 (79%)	232 (25%)	466 (50%)	496 (53%)	243 (26%)	207 (22%)	306 (33%)	220 (23%)
*Regional group 2*		0.080	0.260	0.578	0.415	0.281	0.852	0.873	0.740
Other^l^	n = 98	71 (73%)	17 (17%)	53 (54%)	58 (59%)	31 (32%)	22 (22%)	31 (32%)	23(23%)
Urban * ^m^	n = 1169	934 (80%)	260 (22%)	598 (51%)	642 (55%)	311 (27%)	253 (22%)	379 (32%)	292 (25%)

*Note.* The shaded boxes indicate where the difference in proportions within a subgroup category were statistically significant. ^* ^Auckland, Bay of Plenty, Canterbury, Waikato, and Wellington. Reference groups: ^a^ English as a first language, ^b^Non-English first language, ^c^Permanent visa status, ^d^Temporary visa status, ^e^Non-high school student, ^f^Non-tertiary student, ^g^High school or tertiary student, ^h^No an essential worker, ^i^An essential worker, ^j^Auckland resident, ^k^non-Auckland resident, ^l^urban area resident, ^m^non-urban area resident.

When looking at demographic subgroups in Table 1, significant gender differences were found, with females more likely to obtain COVID-19 information through family and friends compared to males and other gender groups. Significant age differences were also noted, with the oldest age group using most information sources less frequently, except for local ethnic community media and messaging applications, which were used more frequently by this group.

Ethnicity-wise, mainstream media, social media, and government websites were the most used sources, except for the Korean subgroup, which reported high usage of local ethnic community media and lower usage of social media, government websites, search engines, and the workplace. South East Asians used mainstream media, social media, and government websites significantly more. Chinese and Korean respondents reported higher usage of messaging application.

Table 2 reveals that non-English native speakers were significantly less likely to use mainstream New Zealand media, social media, government websites, search engines, family and friends, and the workplace. Conversely, they were significantly more likely to use local ethnic media to obtain COVID-19 related health information.

Regarding student status, all groups primarily used mainstream media, government websites, and social media for COVID-19 information. High school and tertiary students used search engines and family and friends more than non-students, who relied more on local ethnic community media. Essential workers used workplaces more than non-essential workers. In Auckland, ethnic community media usage was higher, whereas government websites, social media, and workplaces were more used outside Auckland.

Table 3 outlines logistic regression findings, indicating that not having English as a first language negatively affected the use of most information sources, except for ethnic community media where the odds of usage were six times higher for non-English speakers than those who have English as a first language, and messaging applications which had a less strong but statistically significant positive association. Overall, these findings suggest that linguistic characteristics can influence both the type of information sources that are accessible and favoured. The association between information sources and ethnicity varied: Chinese participants favoured messaging applications or the workplace, Indian participants preferred government websites, Korean participants relied on ethnic community media, and South East Asian participants were more likely to use mainstream media, government websites, and search engines.

**Table 3:** Logistic Regression of Not Having English as a First Language/ethnic Grouping and Use of Different Information Sources.

Information source	OR	p-value	SE	95% CI
*Mainstream* ***NZ*** *media*				
Not having English as a first language^a^	0.35	0.000	0.07	0.24-0.52
Chinese^b^	1.11	0.522	0.18	0.80-1.53
Indian^c^	0.72	0.166	0.17	0.45-1.15
Korean^d^	0.84	0.228	0.12	0.62-1.12
South East Asian^e^	1.74	0.044	0.48	1.02-2.98
*Ethnic community media*				
Not having English as a first language	6.08	0.000	1.49	3.76-9.82
Chinese	1.01	0.950	0.17	0.73-1.39
Indian	0.22	0.000	0.09	0.10-0.49
Korean	3.33	0.000	0.51	2.47-4.50
South East Asian	0.17	0.000	0.07	0.07-0.39
***NZ*** *government websites*				
Not having English as a first language	0.37	0.000	0.05	0.28-0.47
Chinese	1.05	0.725	0.14	0.80-1.36
Indian	2.03	0.001	0.44	1.34-3.10
Korean	0.47	0.000	0.61	0.37-0.61
South East Asian	1.98	0.000	0.38	1.36-2.90
*Social media*				
Not having English as a first language	0.53	0.000	0.07	0.41-0.69
Chinese	1.07	0.637	0.15	0.82-1.40
Indian	1.36	0.155	0.30	0.89-2.08
Korean	0.51	0.000	0.07	0.39-0.66
South East Asian	2.03	0.001	0.42	1.35-3.06
*Search engines*				
Not having English as a first language	0.39	0.000	0.05	0.30-0.51
Chinese	1.25	0.146	0.19	0.93-1.68
Indian	1.48	0.085	0.33	0.95-2.30
Korean	0.46	0.000	0.08	0.33-0.65
South East Asian	1.63	0.014	0.32	1.10-2.40
*Messaging applications*				
Not having English as a first language	1.31	0.081	0.21	0.97-1.79
Chinese	1.86	0.000	0.28	1.39-2.49
Indian	0.63	0.095	0.17	0.37-1.08
Korean	1.10	0.516	0.16	0.82-1.48
South East Asian	0.44	0.002	0.12	0.26-0.75
*Family, whānau or friends*				
Not having English as a first language	0.72	0.013	0.09	0.56-0.93
Chinese	1.18	0.239	0.16	0.90-1.54
Indian	0.60	0.029	0.14	0.37-0.95
Korean	1.13	0.367	0.16	0.86-1.48
South East Asian	1.02	0.933	0.19	0.70-1.48
*Workplace*				
Not having English as a first language	0.5	0.000	0.07	0.40-0.70
Chinese	1.48	0.010	0.22	1.10-1.98
Indian	1.23	0.361	0.28	0.79-1.93
Korean	0.45	0.000	0.07	0.33-0.63
South East Asian	1.26	0.262	0.26	0.84-1.88

*Note.* The shaded rows indicate where the difference in proportions within a subgroup category were statistically significant. OR =  odds ratio, SE =  standard error. In regression analysis, adjustments were made for age, student status, gender and ethnicity. Reference groups: ^a^English as a first language, ^b^Non-Chinese, ^c^Non-Indian, ^d^Non- Korean, ^e^Non-Southeast Asian.

### Trust in information sources

**Fig 1:**
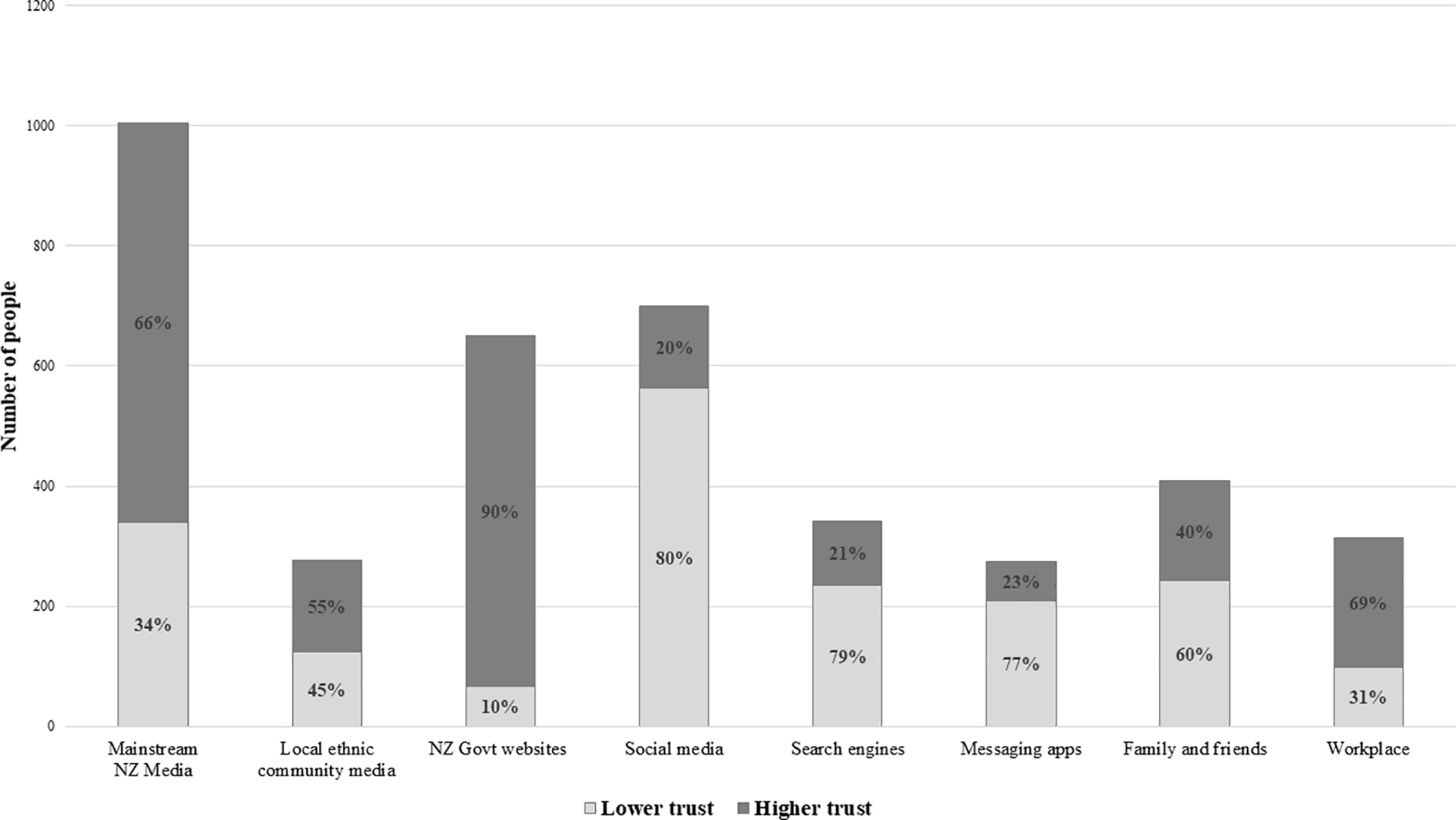
Comparison of the number of respondents who used each information source alongside the proportion who trust/distrust the source. Note. The height of each bar represents the number of respondents who used each information source, while the two patterns within each bar represent the proportions of respondents who trust or distrust the source. The numbers represent the count of respondents who indicated using each information channel to obtain COVID-19 related information. Lower trust refers to those who indicated either no trust, little trust, or some trust in each information source; higher trust refers to those who indicated either lots of trust or complete trust.

Fig 1 compares the number of respondents who used each COVID-related information source with the level of trust they expressed. The highest proportion of respondents in the higher trust category was for government websites, followed by workplace, mainstream New Zealand media, and local ethnic community media. Full descriptive data about the distribution of trust among study participants is available as S1 Table.

Among the eight information sources considered, mainstream New Zealand media, and government websites were the most trusted. Although only a quarter of the total respondents obtained COVID-related information through the workplace, nearly seven out of ten respondents expressed high trust in this source, underscoring the crucial role workplace can play in disseminating health-related information. Additionally, while only around two out of the ten participants used local ethnic community media for COVID information, half of them expressed high trust in this source.

Conversely, social media, which was the second most commonly used source, had eight out of ten respondents expressing lower trust in the COVID information obtained through this channel. Similarly, search engines or messaging applications received lower trust in the COVID information from the participants. Notably, nearly six out of ten respondents expressed lower trust in COVID-related information acquired through close human networks, such as family or friends.

The full findings of linear regression modelling of the association between trust in different information sources and language status/ethnicity group are available in Table 4. The key statistically significant findings were a slightly increase in trust in social media among those who did not have English as a first language and messaging applications for Korean participants. A decrease in trust was found in messaging applications for Chinese participants and information from the workplace for Korean participants.

**Table 4:** Level of trust in different information sources by English language as a first language status and ethnicity: Results from linear regressions.

Information source	p-value of overall model	Coefficient	SE	p-value of coefficient	95% CI
*Mainstream* ***NZ*** *media*					
Not having English as a first language^a^	0.83	0.02	0.10	0.874	-0.19 ~ 0.21
Chinese^b^	0.26	-0.04	0.06	0.469	-0.16 ~ 0.07
Indian^c^	0.28	-0.05	0.10	0.601	-0.24 ~ 0.14
Korean^d^	0.30	0.01	0.06	0.842	-0.10 ~ 0.13
South East Asian^e^	0.25	0.07	0.08	0.417	-0.09 ~ 0.22
*Ethnic community media*					
Not having English as a first language	0.48	0.03	0.31	0.915	-0.59 ~ 0.66
Chinese	0.00	-0.08	0.12	0.497	-0.31 ~ 0.15
Indian	0.00	0.27	0.32	0.403	-0.36 ~ 0.90
Korean	0.00	0.15	0.10	0.146	-0.05 ~ 0.36
South East Asian	0.00	-0.29	0.35	0.412	-0.96 ~ 0.40
***NZ*** *government websites*					
Not having English as a first language	0.00	0.11	0.09	0.206	-0.06 ~ 0.29
Chinese	0.01	-0.04	0.07	0.526	-0.17 ~ 0.09
Indian	0.01	0.14	0.09	0.124	-0.04 ~ 0.32
Korean	0.01	-0.05	0.07	0.487	-0.19 ~ 0.09
South East Asian	0.01	-0.06	0.08	0.473	-0.22 ~ 0.10
*Social media*					
Not having English as a first language	0.19	-0.19	0.09	0.042	-0.38 ~ -0.01
Chinese	0.36	-0.06	0.07	0.362	-0.20 ~ 0.07
Indian	0.36	0.92	0.10	0.361	-0.11 ~ 0.29
Korean	0.45	0.01	0.07	0.839	-0.13 ~ 0.16
South East Asian	0.43	0.04	0.09	0.656	-0.13 ~ 0.20
*Search engines*					
Not having English as a first language	0.58	0.01	0.11	0.923	-0.21 ~ 0.23
Chinese	0.46	-0.11	0.09	0.236	-0.29 ~ 0.07
Indian	0.59	0.09	0.13	0.479	-0.17 ~ 0.36
Korean	0.44	0.14	0.11	0.217	-0.08 ~ 0.36
South East Asian	0.43	0.15	0.12	0.207	-0.08 ~ 0.37
*Messaging applications*					
Not having English as a first language	0.08	-0.01	0.18	0.947	-0.36 ~ 0.34
Chinese	0.01	-0.24	0.10	0.021	-0.44 ~ -0.04
Indian	0.04	0.16	0.21	0.442	-0.25 ~ 0.56
Korean	0.00	0.33	0.10	0.002	0.12 ~ 0.54
South East Asian	0.05	-0.08	0.21	0.701	-0.50 ~ 0.34
*Family, whānau or friends*					
Not having English as a first language	0.04	-0.02	0.13	0.880	-0.27 ~ 0.23
Chinese	0.15	-0.12	0.09	0.160	-0.29 ~ 0.05
Indian	0.27	0.07	0.16	0.670	-0.24 ~ 0.38
Korean	0.14	0.13	0.09	0.144	-0.04 ~ 0.30
South East Asian	0.29	0.02	0.12	0.870	-0.21 ~ 0.25
*Workplace*					
Not having English as a first language	0.45	0.09	0.16	0.551	-0.22 ~ 0.40
Chinese	0.36	-0.04	0.11	0.695	-0.26 ~ 0.17
Indian	0.24	0.19	0.16	0.240	-0.13 ~ 0.52
Korean	0.03	-0.33	0.13	0.010	-0.59 ~ -0.80
South East Asian	0.17	0.22	0.14	0.116	-0.06 ~ 0.50

*Note.* The shaded rows indicate where the difference in proportions within a subgroup category were statistically significant. In regression analysis, adjustments were made for age, student status, gender and English as a first language status. Reference groups: ^a^English as a first language, ^b^Non-Chinese, ^c^Non-Indian, ^d^Non- Korean, ^e^Non-Southeast Asian.

In summary, Asian people living in New Zealand during the first 18 months of the COVID-19 pandemic exhibited high trust in information accessed through formal institutions such as government websites, workplaces, and mainstream New Zealand media. However, there was lower trust in information sources shared online or within personal networks. The regression modelling results further indicated that language status and ethnicity significantly influenced the levels of trust in various information sources, highlighting the need for culturally and linguistically tailored communication strategies.

## Discussions

Our paper provides a unique perspective into the generally lauded New Zealand government’s communication efforts during the first 18 months of the COVID-19 pandemic. By focusing on the experiences of Asian communities, we were able to gain a better understanding of how these communities which include those with diverse migration, language and cultural characteristics described under the label of CALD, sought vital, life-saving information, and how much trust they placed in these sources.

### Access to the information channels among the CALD Asian communities

The current study examined how CALD Asian communities accessed COVID-19 information generated by the New Zealand government. Government interventions for these communities were notably delayed, with linguistically appropriate resources for Asian communities gaining wide attention only by March 2021 [28,29]. Despite this delay, this study identified that CALD Asian communities were actively engaging with a diverse range of information sources.

Among the information channels, mainstream New Zealand media, social media, and government websites were notably more commonly used. When exploring the complexities surrounding information access, we noted specific trends among different age groups. Older individuals predominantly relied on local ethnic community media and messaging applications. A qualitative study of older Korean and Chinese migrants in New Zealand also highlighted limited linguistically appropriate resources as a barrier to understanding COVID-19 rules for this cohort [41]. This trend appears to be linked to a lower proportion of individuals for whom English is a first language. This is supported by demographic analysis of the Asian population of the northern region of New Zealand, which found that older age groups have a higher proportion of people who could not have a conversation in English when compared to older age groups [13]. Access to digital spaces may also be a barrier for some older adults, particularly those who are former refugees or marginalised migrants [42]. Targeted interventions that cater to older adults in CALD communities are necessary to ensure they are not excluded from vital information.

In contrast, younger individuals, particularly those in their teens and twenties, utilised a broader range of information sources. While this diversity allows for a comprehensive view, it also poses challenges in discerning accurate information and exposure to mis- and disinformation [43], especially when the sources include less trusted platforms like social media and messaging applications. Understanding that language and digital access are potential barriers to accessing information and raising greater awareness of the quality of information available through less regulated sources such as social media are both important considerations for governments designing a CALD community crisis communication strategy.

Interestingly, among ethnic subgroups, Korean respondents showed a higher tendency to rely on local ethnic media compared to other ethnic groups. This is a divergence from the general trend observed in other Asian communities, which showed lower usage of local ethnic community media. One explanation for this observation is the consistency and timeliness of information provided by the collaborative efforts of the Korean community and related media organisations during this time. Specifically, these efforts involved a network of volunteers translating government live briefings and disseminating them through online local ethnic community media channels within hours of the English versions, particularly during Level 3 and Level 4 lockdowns [30]. This case illustrates the potential benefits of community-led initiatives and the importance of supporting such networks to enhance crisis communication.

Our findings indicate that non-English speakers were likely to have limited access to information channels compared to English speakers. The exception to this trend was local ethnic community media. This disparity underscores the importance of delivering health information in multiple languages. Proactive development and dissemination of multilingual resources are essential to ensure inclusivity and trust.

### Trust in information sources

This study explored how CALD Asian communities placed their levels of trust in various information sources for accessing COVID-19 government information. Our results underscore the higher levels of trust placed in formal institutions such as the government’s websites, workplaces, mainstream New Zealand media, and local ethnic community media. Conversely, there was significant scepticism towards information acquired through online platforms, including social media, search engines, and messaging applications, as well as through personal networks like family and friends.

These findings provide insights into the opportunities and challenges inherent in disseminating public health information to CALD communities. First of all, government websites not only showed frequent usage but also garnered the highest level of trust among the study participants, despite the limitations to the non-English language information available on this platform in terms of timelines, breadth, and consistency [27]. This emphasises the need for timely, consistent, and multi-language health information via such trusted platforms. Leveraging networks of the embassies and consulates of the CALD Asian communities in New Zealand could facilitate the translation and dissemination of information during crises.

Our research also shows that social media, despite being frequently used by respondents, displayed the lowest levels of trust. This underscores the importance of strategically utilising official social media channels of trusted entities, such as government websites, mainstream media, and local ethnic community media, to enhance credibility of the information on these online platforms.

Despite the high levels of trust in workplace as an information source, they are underutilised. This discrepancy highlights the need for more efforts to make workplaces a primary channel in the government’s crisis communication strategy. In the event of future crises akin to COVID-19, when official health information is generated by the Ministry of Health, a system needs to be established through the New Zealand Ministry of Business, Innovation and Employment to disseminate official and fit-for-purpose health information not only to large enterprises but also to small and medium-sized businesses.

Local ethnic community media emerged as a notably reliable source, while also being one of the few information sources that are linguistically and culturally appropriate for specific ethnic communities [44]. Considering that having a sense of familiarity also makes it easier to spread health information [2], this emphasises the critical role that local ethnic media can play in disseminating reliable health information to these specific demographic groups. Given their operational challenges, supporting these outlets through funding and providing translated materials are practical ways for the governments to enhance their capacity as trusted information channels for CALD communities during crises.

In conclusion, amidst the challenges posed by the infodemic—a surge in misinformation and disinformation [45]—it becomes paramount to underscore the significance of relying on trusted information sources. The insights from this study hold particular significance in advocating for the promotion and utilisation of these reliable sources as an essential step in safeguarding public health within CALD communities.

### Implications for policy

To improve crisis communication strategies, it is essential to recognise the diverse needs of CALD communities. Through this research, we have gained significant insights into the complexities of how sub-groups within CALD Asian communities in New Zealand access and trust various information channels during public health crises. This understanding prompts several critical considerations for future crisis communication strategies.

Efficient routes for disseminating information to non-English speaking members of CALD communities, as well as the most appropriate route for subgroups such as older adults, students, workers, and newer migrants should be identified and can utilised in a more specific manner. For instance, using popular messaging applications like WeChat for the Chinese community could be advantageous, given their social media-like functionalities and our finding that not having English as a first language was associated with higher use of this route. These insights could also inform targeted information for high priority groups, such as older Chinese and Indian adults who were identified as populations with disproportionately low rates of COVID-19 booster vaccination [46,47]. Such tailored campaigns would require the government to foster a workforce with the linguistic and cultural skills to perform this work, or form and maintain relationships with community organisations who can.

Our study also reveals that alongside significant proportions of CALD Asian communities who prefer to communicate in a language other than English, older individuals often rely on local ethnic community media and may require resources translated into their native languages. This observation highlights the need for linguistically appropriate materials to be made available, especially for those with limited access to digital platforms. Given that members of this subgroup were highlighted as a ‘hard-to-reach’ group during the COVID-19 vaccine rollout [48], efforts to improve information delivery to this group should be considered a high priority.

Governments should develop and maintain multilingual resources, leverage trusted community channels for information dissemination, address the digital divide through targeted interventions and media literacy programs, and support community-led initiatives to ensure timely and culturally appropriate information dissemination. In terms of delivering trusted information, mainstream media remains crucial but needs to be available both online and offline and in languages appropriate to specific communities. Since this study was conducted, one mainstream media outlet in New Zealand has made developments in this space, with Radio New Zealand launching an initiative to better cater to Chinese and Indian communities [49]. Similarly, government websites, which our study shows are highly trusted, should be easily accessible, provide timely information and offer multilingual support. Workplaces, though highly trusted, are underutilised and should be considered for targeted dissemination strategies, particularly for the working population and their households.

By implementing these strategies, governments can ensure that all members of CALD communities have access to vital information during crises, ultimately enhancing public health outcomes and community resilience. Finally, it is essential for the government to undertake proactive measures before the next crisis occurs. Although a large-scale crisis such as a global pandemic is unlikely to happen frequently, the strategies and capacity developed to deliver information effectively to CALD communities will also be useful for more common situations such as localised communicable disease outbreaks and civil defence events. To ensure timely access to translated materials, a pre-existing network that can distribute linguistically and culturally appropriate information must be established. While some communities, such as the Korean community, have independently translated and distributed government briefings [50], relying solely on these self-organised efforts can exacerbate inequalities among communities with varying levels of resources and are unlikely to be easily replicated during future crises. To mitigate this, it is imperative that a dedicated budget and workforce be allocated to support these distribution networks in times of crisis.

### Limitations and recommendations for future studies

This study provides novel primary data for understanding the challenges and opportunities of crisis communication within CALD Asian communities in New Zealand. However, it comes with limitations that necessitate further exploration in subsequent studies.

One significant limitation pertains to the use of non-probabilistic purposive and snowball sampling techniques. These methods, while efficient under the constraints of time and budget, may introduce selection bias and limit the generalisability of the findings. The non-probabilistic approach means that our sample may not fully represent the wider population, particularly those who were harder to reach due to the lockdown and digital divide. In particular, our online-centric data collection approach led to lower participation rates among individuals aged 60 and above, and potentially other groups who have less digital access. This gap in our study suggests that the needs of older members within CALD communities, who may be less adept at navigating digital spaces, could be underrepresented. Therefore, a crucial avenue for future research involves utilising probabilistic sampling methods and integrating both online and offline recruitment strategies, especially in collaboration with community organisations, which can provide more comprehensive insights into the information-seeking behaviours of older individuals who are less comfortable in digital spaces.

Moreover, the study did not explore the government’s actions, resourcing allocation or decision-making processes around making crisis information accessible to CALD communities nor did it investigate how much these communities’ perspectives are reflected in crisis communication policies. This opens up a new dimension for future research—evaluating existing policies and resources to identify both strengths and areas for improvement. Such an assessment could provide actionable recommendations for making government communication strategies more inclusive and effective.

Additionally, while the term CALD community serves as an inclusive and non-stigmatizing umbrella term, it inherently encompasses a diverse array of subgroups with unique characteristics and challenges. Our study primarily focuses on the CALD Asian community in New Zealand, with particular emphasis on the four largest Asian ethnic groupings: Chinese, Indian, Korean, and Southeast Asian communities. This focus means that our findings primarily reflect the experiences and perspectives of these subgroups and may not fully represent the diversity within the entire CALD Asian community. Future research should aim to include a more comprehensive range of CALD subgroups to provide a more nuanced understanding of their unique experiences and needs.

While the study does provide a window into the CALD Asian communities in New Zealand, it does not offer a panoramic view of all CALD communities in the country. Future research could broaden the scope to include other CALD communities, examining their unique information-seeking behaviours and trust levels. Two groups that intersect with CALD communities, who have had unique experiences during the pandemic and are often excluded in designing policy, are those from a refugee or asylum-seeker background [51], and low-income communities [52]. The perspectives of groups such as these would offer a more holistic understanding of the challenges and opportunities in crisis communication for CALD communities at large, as well as key groups who may require specialised outreach or support.

In addition, our study did not delve into the linguistic and cultural appropriateness of the crisis information provided. Subsequent research could include community-level case studies during crises like the COVID-19 pandemic to assess the effectiveness of these communication efforts. Such an analysis could offer a nuanced understanding of how well current communication strategies meet the unique needs of CALD communities.

Finally, while we did investigate the trust levels associated with different information sources, we did not specifically look at the trust placed in government-provided information during a crisis. Understanding this could be pivotal for future communication strategies, particularly in times of public health emergencies and in the context of disinformation being a powerful factor shaping people’s perspectives and beliefs.

In summary, this study lays the groundwork for future research in a largely unexplored area. The limitations identified herein are not just constraints but also opportunities for future research to build upon. They offer multiple avenues for deeper, more comprehensive studies that can inform more effective and inclusive crisis communication strategies. We posit that in the face of health and civil defence emergencies, it is the duty of health and other government agencies to ensure that everyone has access to crucial information. Our findings indicate that a singular approach to crisis communications may not suffice for diverse communities. Beyond merely highlighting these challenges, our research offers actionable insights that are not only valuable for understanding the current situation but can also guide health and other governmental agencies in strengthening communication networks for future crises.

## Supporting information

S1 Table Overall level of trust in information sources (%)
(DOCX)
