# Supplementary material for: Examining access to and trust in sources of COVID-19 information among CALD Asian communities in New Zealand
Source: PLoS One. 2025 Mar 28;20(3):e0319326. doi: 10.1371/journal.pone.0319326 (PMC11952248; doi:10.1371/journal.pone.0319326)
Supplement: S1 Table — (DOCX) [file pone.0319326.s001.docx]

**S1 Table. Overall level of trust in information sources (%)**

| Trust  level | Mainstream NZ Media | Local ethnic community media | NZ Govt websites | Social Media | Search Engines | Messaging apps | Family and friends | Workplace |
| --- | --- | --- | --- | --- | --- | --- | --- | --- |
|  | n=1005 | n=276 | n=651 | n=700 | n=342 | n=274 | n=409 | n=315 |
| No trust | 1% | 1% | 0% | 2% | 1% | 3% | 0% | 0% |
| Little trust | 4% | 8% | 1% | 22% | 9% | 22% | 8% | 6% |
| Some trust | 29% | 36% | 9% | 56% | 69% | 52% | 52% | 25% |
| ***Total***  ***(Lower trust)*** | 34% | 45% | 10% | 80% | 79% | 77% | 60% | 31% |
| Lots of trust | 45% | 43% | 41% | 16% | 27% | 20% | 32% | 44% |
| Complete trust | 21% | 12% | 49% | 4% | 5% | 4% | 9% | 24% |
| ***Total***  ***(Higher trust)*** | 66% | 55% | 90% | 20% | 21% | 23% | 40% | 69% |
